# Engineering Topological Spin Hall Effect in 2D Multiferroic Material

**DOI:** 10.1002/advs.202407982

**Published:** 2024-10-01

**Authors:** Kaiying Dou, Zhonglin He, Jiangyu Zhao, Wenhui Du, Ying Dai, Baibiao Huang, Yandong Ma

**Affiliations:** ^1^ School of Physics State Key Laboratory of Crystal Materials Shandong University Shandanan Str. 27 Jinan 250100 P. R. China

**Keywords:** 2D multiferroic material, antiferromagnetic bimeron, ferroelectricity, first‐principles, topological spin Hall effect

## Abstract

Topological spin Hall effect (TSHE), promoted by coupling between noncoplanar spins and real‐space topology, is a significant phenomenon in condensed matter physics. However, the control of TSHE characteristics is missing due to its intrinsic robustness, and such fundamental difficulty prevents it from being used for spintronics up to now. Here, a rational design approach is demonstrated to engineer TSHE in a controllable and reversible fashion. Through symmetry and model analysis, it is unveiled that antiferromagnetic topological charge, as well as Lorentz forces, acted on conduction electrons, can be coupled with Dzyaloshinskii‐Moriya interaction chirality for antiferromagnetic bimerons in 2D multiferroic materials. Such coupling guarantees the ferroelectric control of TSHE. Using first‐principles calculations and atomic spin model simulations, the validity of this mechanism is further demonstrated in multiferroic monolayer CuCr_2_Se_4_ with experimental feasibility. The alter‐chirality of the Dzyaloshinskii‐Moriya interaction is found to play a crucial role in realizing this mechanism. This results extend TSHE to be used in spintronics and open a new direction for spintronics research.

## Graphical abstract

Antiferromagnetic topological charge, as well as Lorentz forces acting on conduction electrons, can be coupled with Dzyaloshinskii‐Moriya interaction chirality for antiferromagnetic bimerons in multiferroic lattice. Such coupling guarantees the engineer of topological spin Hall effect in a controllable and reversible fashion.

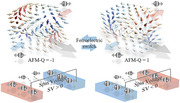

## 1 Introduction

Topological magnetism is localized swirling spin textures exhibiting nontrivial real‐space topology,^[^ ^1^, ^2^, ^3^, ^4^ ^]^ which have been investigated intensively due to a variety of exotic characteristics.^[^ ^5^, ^6^, ^7^, ^8^ ^]^ In ferromagnets, these chiral spin configurations can induce a transverse deflection of itinerant electrons known as the topological Hall effect.^[^ ^9^, ^10^, ^11^ ^]^ Meanwhile, such chiral spin configurations themselves also experience a deflection transverse to the current, which is referred to as skyrmion Hall effect (SkHE).^[^ ^12^, ^13^, ^14^ ^]^ In antiferromagnetic (AFM) topological configurations, the SkHE can be inhibited because of the opposite Magnus force of each spin sublattice;^[^ ^15^, ^16^ ^]^ whereas, the Lorentz forces transversely deflect the spin‐up and spin‐down flowing electrons to opposite directions, thereby generating the concept of topological spin Hall effect (TSHE).^[^ ^17^, ^18^, ^19^, ^20^]

As an emerging unique member of the Hall family, TSHE is of significant fundamental in condensed‐matter physics.^[^ ^20^, ^21^ ^]^ In principle, with such a compelling nature, TSHE should claim a prominent place in the realm of future spintronics applications. To this end, it is essential to achieve effective control of TSHE characteristics. Nevertheless, as TSHE is rooted in AFM topological systems,^[^ ^17^ ^]^ it is insensitive to external perturbations and thus intrinsically robust, which makes its efficient manipulation challenging. This is in sharp contrast to the ordinary Hall effects, wherein various engineering strategies (e.g., magnetic field, electric field, circularly polarized light) are well established.^[^ ^22^, ^23^ ^]^ As a result, although TSHE has been firmly established through numerous efforts, it has not yet found its transport device applications and currently is only utilized to detect the emergence of AFM topological magnetism.^[^ ^24^, ^25^ ^]^ Actually, up to now, it is still unclear how to control the TSHE characteristics.

In this work, using symmetry and model analysis, we propose a general design approach for realizing the effective control of TSHE in a controllable and reversible fashion. We reveal that, for antiferromagnetic bimerons in 2D multiferroic materials, the antiferromagnetic topological charge and Lorentz forces acted on conduction electrons can closely correlate to Dzyaloshinskii‐Moriya interaction (DMI) chirality. This enables the reversal of topological spin Hall conductivity through ferroelectric (FE) switching, thereby realizing the ferroelectrically control of TSHE. Based on first‐principles calculations and atomic spin model simulations, we further demonstrate the validity of this mechanism in a multiferroic lattice of monolayer CuCr_2_Se_4_, which possesses alter‐chiral DMI and has been synthesized in the experiment.^[^ ^26^, ^27^, ^28^ ^]^ We show that the alter‐chirality of DMI for the multiferroic lattice plays a crucial role in realizing this mechanism. The underlying physics is discussed. These findings greatly enrich the research on TSHE and spintronics.

## 2 Results

**Figure 1:**
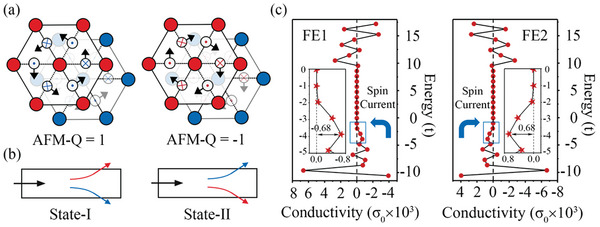
a) Schematic diagrams for AFM lattices with opposite AFM‐Q under DMI chirality reversal. b) Schematic diagrams for two states of TSHE. The red and blue arrows in b) illustrate the deflection directions of spin‐up and spin‐down polarized conduction electrons, respectively. c) Topological spin Hall conductivities for FE1 and FE2. Blue arrows in c) indicate deflection directions of spin currents from conduction electrons around the Fermi level. In (c), *σ*_*0*_ =  e/4π and *t* = *t_2_* = 0.04 in the Hamiltonian of the tight‐binding model.

The proposed mechanism is based on 2D antiferromagnetic (AFM) multiferroic hexagonal lattice with two sublattices carrying alternating magnetic moments (as illustrated in the left panel of **Figure** **1**a). For the AFM bimeron generated in such lattice, the spin textures can be described by the normalized staggered order vector of ***n*** (***r***)* * =  [***M**_A_* (***r***) − ***M**_B_* (***r***)]/2, where ***M**_A_* (***r***) and ***M**_B_* (***r***) are the normalized local magnetic moments of sublattice A and B, respectively.^[^ ^20^ ^]^ The interaction between the spin texture and current can be taken as equivalent electromagnetic fields acted on conduction electrons:^[^ ^21^, ^26^ ^]^

(1) $$E_{\sigma }^{\mu }=\frac{\sigma \hbar }{2e}\;P_{\sigma }^{\mu }N_{t,i}\left(r\right)e_{i}$$

(2) $$B_{\sigma }^{\mu }=-\frac{\sigma \hbar }{2e}P_{\sigma }^{\mu }N_{x,y}\left(r\right)z$$

with

(3) $$N_{j,k}\left(r\right)=n\left(r\right)\cdot \left(\frac{\partial n\left(r\right)}{\partial j}\times \frac{\partial n\left(r\right)}{\partial k}\right)\;j,k\in \left\{t,x,y\right\}$$

$P_{\sigma }^{\mu }$ is defined as $P_{\sigma }^{\mu }=\frac{1}{2}\;\left[1+\sigma \mu P(k)\right]$, which couples the polarization density of states *P*(*k*) with spin index σ (σ = +/− 1 for spin‐up (σ_+_)/spin‐down (σ_−_) conduction electrons) and sublattice index μ (μ = +/− 1 for sublattice A/B). *P*(*k*) can be expressed as^[^ ^21^, ^26^, ^27^ ^]^

(4) $$P\left(k\right)=\frac{J}{\sqrt{{\left|\gamma \left(k\right)\right|}^{2}+J^{2}}}$$

Here, *J* represents the coupling between conduction electron and spin texture ***n*** (***r***). γ(*k*) indicates the nearest‐neighboring hopping, which is proportional to the nearest‐neighboring Heisenberg exchange energy *t*. When separating the two sublattices with a relatively large space, *t* would be significantly weakened. Then, as compared with *t*, *J* can be considered to infinity. This results in *P*(*k*) → 1, corresponding to that all conduction electrons tend to align parallel to the background texture profile.^[^ ^28^, ^29^ ^]^ As a result, the following relations can be obtained as

(5) $$\begin{array}{cc} \mu \;=\;1 & \left\{\begin{array}{cc} \sigma \;=\;1 & \;P_{\sigma_{+}}^{A}=\;1\\ \sigma \;=\;-1 & \;P_{\sigma_{-}}^{A}=\;0 \end{array}\right. \end{array}\;\begin{array}{cc} \mu \;=\;-1 & \left\{\begin{array}{cc} \sigma \;=\;1 & \;P_{\sigma_{+}}^{B}=\;0\\ \sigma \;=\;-1 & \;P_{\sigma_{-}}^{B}=\;1 \end{array}\right. \end{array}$$

and

(6) $$\begin{array}{cc} \;E_{\sigma_{+}}^{A}=\frac{\hbar }{2e}\;N_{t,i}\left(r\right)e_{i} & \;E_{\sigma_{-}}^{A}=\;0\\ \;B_{\sigma_{+}}^{A}=\;-\frac{\hbar }{2e}N_{x,y}\left(r\right)z & \;B_{\sigma_{-}}^{A}=\;0 \end{array}\;\begin{array}{cc} \;E_{\sigma_{-}}^{B}=\;-\frac{\hbar }{2e}N_{t,i}\left(r\right)e_{i} & \;E_{\sigma_{+}}^{B}=\;0\\ \;B_{\sigma_{-}}^{B}=\;\frac{\hbar }{2e}N_{x,y}\left(r\right)z & \;B_{\sigma_{+}}^{B}=\;0 \end{array}$$

The physical origin of the electrons deflection predominantly stems from the emergent magnetic field,^[^ ^21^, ^30^, ^31^ ^]^ which exerts a Lorentz force $F=e\left(\dot{r}\times B\right)$ on conduction electrons. Therefore, we obtain $F_{\sigma_{+}}^{A}=\;-F_{\sigma_{-}}^{B}$. This indicates that the Lorentz force experienced by spin‐up electrons in sublattice A is exactly opposite to that experienced by spin‐down electrons in sublattice B, leading to the topological spin Hall effect (TSHE). It is worth pointing out that the relatively large separation between the two sublattices is essential for realizing the significant TSHE.

**Figure 2:**
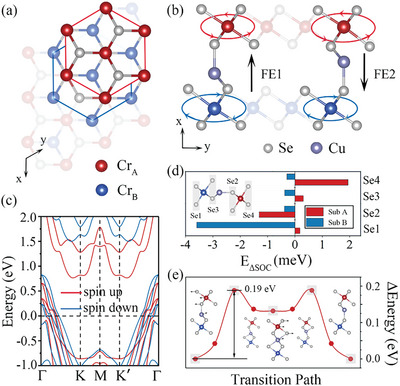
a) Top and b) side views of the crystal structure of monolayer CuCr_2_Se_4_. Red and blue hexagons in (a) indicate the sublattices A and B, respectively. In (b), the arrows and size of circles represent the DMI chirality and strength, respectively. c) Spin‐polarized band structures of monolayer CuCr_2_Se_4_. d) Atomic‐resolved localization of DMI‐associated SOC energy. In d), the positive (negative) value donates the inclination of anticlockwise (clockwise) DMI. e) Ferroelectric transition pathway and energy barrier between FE1 and FE2.

From above, we can see that the Lorentz forces acted on conduction electrons in such AFM bimeron is determined by *N*_*x*,*y*_(***r***). Note that the expression of *N*_*x*,*y*_(***r***) is similar to AFM topological charge $\mathrm{AFM}-Q=\frac{1}{4\pi }\int n\left(r\right)\cdot \left(\frac{\partial n(r)}{\partial x}\times \frac{\partial n(r)}{\partial y}\right)dxdy$, resulted in the Lorentz forces locked with AFM‐Q. It has a similar expression to the topological charge Q in conventional ferromagnetic (FM) topological magnetism, but the spin texture is described as ***n*** (***r***) = [***M**_A_* (***r***) − ***M**_B_* (***r***)]/2.^[^ ^20^, ^26^ ^]^ AFM‐Q can be understood as the winding number of a set of AFM spin pairs in topological magnetism, corresponding to the FM topological charge Q.^[^ ^32^ ^]^ In principle, according to the definition of AFM‐Q, it can be inversed through spin orientation reversal as shown in Figure S2a (Supporting Information). This strategy, however, is impractical for such AFM coupling systems as they are insensitive to external magnetic fields.^[^ ^33^, ^34^ ^]^ Intriguingly, we find that the AFM‐Q of such AFM bimeron can also be reversed by Dzyaloshinskii‐Moriya interaction (DMI) chirality reversal as shown in **Figure** **2**a and S2b (Supporting Information). This is in sharp contrast to the case of skyrmion wherein DMI chirality does not change topological charge as shown in Figure S2b and detailed in Note S4 (Supporting Information).^[^ ^6^, ^35^, ^36^, ^37^ ^]^ Here, we take the AFM bimeron evaluated from Néel‐type AFM skyrmion by rotating all spins by 90° along *y* axis as shown in Figure S3a and detailed in Note S5 (Supporting Information) as an example to illustrate this point. In such AFM bimeron, ***M**_A_* (***r***) and ***M**_B_* (***r***) in polar coordination (*r*,  θ) can be represented as

(7) $$M_{A}\left(r,\theta \right)=\left(cos\;r,\;sin\;r\cdot sin\;\theta ,\;-sin\;r\cdot cos\;\theta \right)$$

(8) $$M_{B}\left(r,\theta \right)=\left(-cos\;r,\;-sin\;r\cdot sin\;\theta ,\;sin\;r\cdot cos\;\theta \right)$$

When switching the DMI chirality, ***M**_A_* (***r***) and ***M**_B_* (***r***) are changed to (the right panel of Figure S3d and detailed in Note S5 (Supporting Information)

(9) $$M_{A}{\left(r,\theta \right)}^{\prime }=\left(cos\;r,\;sin\;r\cdot sin\;\theta ,\;sin\;r\cdot cos\;\theta \right)$$

(10) $$M_{B}{\left(r,\theta \right)}^{\prime }=\left(-cos\;r,\;-\;sin\;r\cdot sin\;\theta ,\;-sin\;r\cdot cos\;\theta \right)$$

In this sense, the AFM‐Q is reversed based on its expression. As a result, the DMI chirality reversal can reverse the Lorentz forces acted on conduction electrons, i.e., the deflection directions of the polarized electrons are reversed (as illustrated in Figure 1b). As the DMI chirality in 2D lattice is locked with local structure symmetry, its reversal can be strongly coupled with ferroelectricity,^[^ ^6^, ^38^, ^39^ ^]^ which suggests the ferroelectric (FE) control of TSHE can be readily realized.

To get more insight into this mechanism, we employ a tight‐binding model for describing the AFM bimerons shown in Figure 1a. The Hamiltonian for the interaction between spin texture and conduction electrons can be described as^[^ ^8^, ^14^ ^]^

(11)

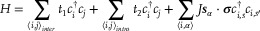

where $c_{i}^{†}\left(c_{j}\right)$ indicates the creation (annihilation) operator and ***σ*** is the vector of Pauli matrices. The inter‐sublattice hopping is quantified by *t*_1_ and the hopping between the nearest neighboring sites in each sublattice is quantified by *t*_2_. *J* indicates the Hund's coupling strength between conduction electron spin and background spin texture.^[^ ^28^, ^29^ ^]^ For simplicity, a 7 × 7 supercell is chosen (each unit cell contains two opposite magnetic moments). The Berry curvature is determined by the eigenvectors *u_nk_* and eigenvalues *E_nk_* as following^[^ ^17^, ^28^ ^]^

(12) $$\Omega_{n}\left(k\right)=i\sum_{m\neq n}\frac{\langle u_{nk}|\nabla_{k}MH_{k}|u_{mk}\rangle \times \langle u_{nk}|\nabla_{k}H_{k}|u_{mk}\rangle }{{\left(E_{nk}-E_{mk}\right)}^{2}}$$

The Matrix M is *diag*(***n*** (**r**_1_) · ***σ***, *** n*** (**r**_2_) · ***σ***, * *⋅⋅⋅, *** n*** (**r**_49_) · ***σ***), which accounts for the alignment of conduction electrons following background bimeron.^[^ ^17^ ^]^ The transverse spin Hall conductivity σ_*xy*_(*E_F_*) is calculated by the Kubo formula^[^ ^17^, ^28^ ^]^

(13) $$\sigma_{xy}\;\left(E_{F}\right)=\frac{\sigma_{0}}{2\pi }\;\sum_{n}\int \Omega_{n}\left(k\right)\cdot f\left(E_{nk}-E_{F}\right)d^{2}k$$

where σ_0_ =  e/4π and *f*(*E_nk_* − *E_F_*) is Fermi‐Dirac distribution function. The corresponding results are shown Figure 1c. For both cases, significant spin Hall conductivity is obtained. More importantly, with reversing the DMI chirality through ferroelectricity, the spin Hall conductivity indeed is reversed, thus firmly confirming the TSHE in such AFM bimeron is ferroelectrically controllable.

One candidate system to realize the proposed mechanism is monolayer CuCr_2_Se_4_, which has been synthesized in the experiment.^[^ ^40^, ^41^, ^42^, ^43^ ^]^ Figure 2a,b present its crystal structure, which exhibit a hexagonal lattice with the space group *P3m1*. The lattice parameter is optimized to be 3.63 Å, agreeing well with previous work.^[^ ^44^, ^45^ ^]^ Each unit cell of monolayer CuCr_2_Se_4_ can be regarded as consisting of two T‐phase CrSe_2_ units separated by an off‐centered Cu atom. The displacement of Cu atom results in two energetically degenerate FE states (i.e., FE1 and FE2, as shown in Figure 2b). For the convenience of discussion, we refer to the Cr atom closer to Cu atom in FE1 state as Cr_B_, and the other Cr atom as Cr_A_. Obviously, Cr_A_ and Cr_B_ atoms belong to sublattices A and B, respectively. The electric polarizations are calculated to be 2.67 and −2.67 µC cm^−2^ for FE1 and FE2 states, respectively. The energy barrier for the ferroelectric transition is feasible due to the barrier of 0.19 eV per formula unit as shown in Figure 2e, which is in good agreement with previous work.^[^ ^45^ ^]^

In addition to ferroelectricity, monolayer CuCr_2_Se_4_ also possesses spin polarization. The magnetic moments contributed by Cr_A_ and Cr_B_ atoms in FE1 state are calculated to be 3.53 and 3.61 *µ_B_*, respectively. By transforming into FE2 state, the absolute values of the magnetic moments on Cr_A_ and Cr_B_ atoms would be reversed, suggesting the intriguing magnetoelectric coupling. The spin‐polarized band structure of monolayer CuCr_2_Se_4_ is shown in Figure 2c, from which we can see that it exhibits a metallic nature. Although the system we discussed is metallic, the mechanism can also be extended to semiconducting counterparts providing the conduction electrons are generated.

To study the interaction among spins of monolayer CuCr_2_Se_4_, we employ a 2D atomic spin Hamiltonian model, which can be written as

(14) $$\begin{array}{ccc} H & = & -\sum_{n}\sum_{{\langle i,j\rangle }_{n}}J_{n}S_{i}\cdot S_{j}-\sum_{m}\sum_{{\langle i,j\rangle }_{m}}J_{m}^{inter}S_{i}\cdot S_{j}-\sum_{l}\sum_{{\langle i,j\rangle }_{l}}d_{l}\cdot \left(S_{i}\times S_{j}\right)\\ & & -K\sum_{i}{\left(S_{i}^{z}\right)}^{2} \end{array}$$

Here, ***S***_*i*(*j*)_ is the normalized unit vector for the actual atomic moment. *J*_*n*_(*n*  =  *A*1, *A*2, *B*1, *B*2) describe the nearest and next‐nearest neighboring Heisenberg exchange couplings in each sublattice (Figure S4b, Supporting Information). $J_{m}^{inter}\left(m\;=\;1,2\right)$ corresponds to the Heisenberg exchange couplings between two sublattices (Figure S4a, Supporting Information). ***d**_l_* = *d_l_* (***u***_*i* → *j*_ × ***z***) (*l*  =  1, 2) indicates the in‐plane DMI between the nearest neighboring sites in the two sublattices. The last term describes the single‐ion anisotropy (SIA).

As listed in **Table** **1**, the magnetic parameters of the two sublattices are not equivalent, which can be attributed to the existence of electric polarization. For sublattice A of FE1, the nearest neighboring exchange parameter *J_A1_* is positive, suggesting the FM coupling. As compared with *J_A1_*, the strength of the next‐nearest neighboring interaction *J_A2_* is significantly weak, and the negative value suggests the AFM coupling. For sublattice B of FE1, *J_B1_* and *J_B2_* exhibit a similar trend, but possess slightly stronger coupling strengths. While for the exchange interaction between two sublattices, $J_{2}^{inter}$ with a negative value is remarkably larger than $J_{1}^{inter}$, indicating a AFM coupling between the two sublattices. In this sense, AFM multiferroics with the two sublattices carrying alternating magnetic moments are identified in monolayer CuCr_2_Se_4_. And intriguingly, the relatively large separation between the two sublattices, as discussed above, would be essential for realizing the significant TSHE.

**Table 1:** Heisenberg exchange interaction parameter *J_n_*, exchange coupling $J_{m}^{inter}$ between sublattice, in‐plane DMI parameter *d_l_* and SIA parameter *K* of monolayer CuCr_2_Se_4_.

	*J*_*A*1_	*J*_*A*2_	*J*_*B*1_	*J*_*B*2_	$J_{1}^{inter}$	$J_{2}^{inter}$	*d*_1_	*d*_2_	*K*
FE1	25.400	−3.706	31.810	−5.423	0.619	−1.755	−0.479	1.563	−0.238
FE2	31.810	−5.423	25.400	−3.706	0.619	−1.755	−1.563	0.479	−0.238

Different from the case of Heisenberg exchange interaction, as listed in Table 1, the DMI chirality of sublattices A and B are opposite, giving rise to an alter‐chiral DMI in monolayer CuCr_2_Se_4_. Moreover, the corresponding DMI strengths are remarkably different in the two sublattices. In FE1, the DMI strength for sublattice B is approximately three times that for sublattice A. As a consequence, the DMI in sublattice B would play a dominant role in forming the noncoplanar spin structure. It should be noted that the alter‐chirality of DMI for the multiferroic lattice plays a crucial role in realizing this mechanism. When stacking into multi‐layer systems and considering the effect of van der Waals gaps, the alter‐chirality of DMI, and thus the mechanism, might be deformed. Additionally, as the alter‐chirality of DMI can be hardly achieved in bulk multiferroic systems, the mechanism proposed here is not expected to be realized in the 3D systems.

To understand such discrepancy in DMI, we calculate the atomic‐resolved localization of DMI‐associated SOC energy. $\Delta \;E_{SOC}=E_{SOC}^{ACW}\;-E_{SOC}^{CW}$. As shown in Figure 2d, the anticlockwise DMI in sublattice A and clockwise DMI in sublattice B primarily originate from Se4 and Se1 atoms, respectively. While Se3 weakens the DMI in sublattice A, all Se atoms strengthen the DMI in sublattice B. With these results in hand, we can understand why the DMI in sublattice B is stronger than that in A. The SIA parameter *K* is found to be −0.238 meV, implying the in‐plane magnetization orientation is preferred.

With transforming FE1 into FE2, the off‐centered Cu atom moves toward Cr_A_ atomic layer, and thus the coordinated environments for Cr_A_ and Cr_B_ are exchanged. Therefore, the strength of the Heisenberg exchange interaction in sublattice A is swapped with that in sublattice B. However, the Heisenberg exchange interactions between the two sublattices remain unchanged, as listed in Table 1. While SIA parameter *K*, it is also robust to FE transition. Meanwhile, the FE transformation swaps the DMI strength of the two sublattices, but their DMI chirality is preserved. In this regard, the dominant DMI in FE1 is from sublattice B (i.e., −0.479 meV in sublattice A and 1.563 meV in sublattice B), while that in FE2 is from sublattice A (i.e., −1.563 meV in sublattice A and 0.479 meV in sublattice B). In other words, the chirality of the dominant DMI to form the noncoplanar spin structure can be ferroelectrically reversed.

**Figure 3:**
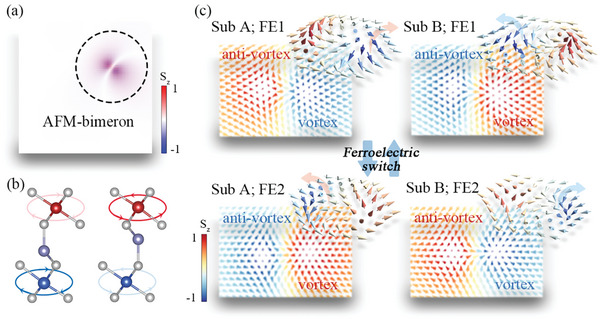
a) Spin texture in FE1 of monolayer CuCr_2_Se_4_. b) Schematic diagrams for chirality of equivalent DMI corresponding to spin textures in sublattices of FE1 (left panel) and FE2 (right panel). Blue and Red circles indicate the dominant DMI for FE1 and FE2, respectively. The light red and light blue circles indicate the equivalent DMI reversed by inter‐sublattice coupling for FE1 and FE2, respectively. c) Spin textures of each sublattice for FE1 and FE2, wherein the large arrows indicate the transverse deflection directions of spin‐polarized conduction electrons.

Based on the first‐principles parameterized Hamiltonian, we perform atomic spin model simulations to explore the spin structures of monolayer CuCr_2_Se_4_. The spin structure for FE1 is displayed in **Figure** **3**a, wherein an AFM bimeron with AFM‐Q = −1 is observed. Figure S5a (Supporting Information) presents the enlarged view of the spin texture, which illustrates the direction of each spin within the AFM bimeron. To explore deeper insights into the AFM bimeron, we separate the spin textures of sublattices A and B for FE1. As shown in the top panel of Figure 3c, the AFM bimeron consists of two FM bimerons with opposite magnetization. Although the magnetic parameters in sublattices A and B are different, the two FM bimerons are almost identical with reversing each spin, which can be attributed to the non‐negligible inter‐sublattice coupling. The FM bimeron in sublattice A is characterized by an anti‐vortex with upward central spin polarization (Q = −1/2; detailed in Note S9 (Supporting Information) and a vortex with downward central spin polarization (Q = −1/2), giving rise to Q of −1. While in sublattice B, it contains an anti‐vortex with downward central spin polarization (Q = 1/2) and a vortex with upward central spin polarization (Q = 1/2), which results in Q = 1.

The spin textures for FE2 are shown in Figure S5b (Supporting Information). It is found that the topological charge of the AFM bimeron is reversed to AFM‐Q = 1. The corresponding FM bimeron in sublattice A/B consists of an anti‐vortex with downward/upward central spin polarization (Q = + /− 1/2) and a vortex with upward/downward central spin polarization (Q = + /− 1/2). For the convenience of discussion, we use the chirality of the vortex in the bimeron to characterize the chirality of bimeron. The anticlockwise (clockwise) chirality of spin textures is observed for FE1 (FE2) state in both sublattices (i.e., the chirality of spin textures in both sublattices are identical for each FE state), which agrees with the chirality of the corresponding dominant DMI (See Figure 3b). Therefore, the DMI chirality and AFM‐Q in monolayer CuCr_2_Se_4_ is strongly coupled with ferroelectricity, implying the TSHE is ferroelectrically controllable.

It should be noted that spin texture chirality presents a discrepancy with the calculated DMI chirality listed in Table 1, which shows opposite chirality for the two sublattices. This discrepancy is related to the joint effect of the inter‐sublattice interactions and the DMI chirality competition between the two sublattices. To further explore this discrepancy, we take FE1 state as an example and artificially vary the inter‐sublattice interactions to study their effects on the spin textures. Figure S6a (Supporting Information) displays the specific spin textures in the sublattices without considering inter‐sublattice interactions (case‐I). It exhibits the bimeron in sublattice A with clockwise chirality and the skyrmion in sublattice B with anticlockwise chirality, which agrees with the DMI chirality listed in Table 1. By reducing the inter‐sublattice interaction to 1/10 of the original strength (case‐II), a novel spin texture is presented in sublattice A with Q = 0 (See the top panel of Figure S6b, Supporting Information). The spin texture is similar to bimeron, but without composing of vortex and anti‐vortex. Instead, it consists of two propagated spin spiral domains with opposite out‐of‐plane components, and the in‐plane component is aligned with the direction of FM background. For the corresponding sublattice B, there is a bimeron with anticlockwise chirality as shown in the bottom panel of Figure S6b (Supporting Information). When the inter‐sublattice interactions are proportionally scaled down to half of their original strength (case‐III), as shown in Figure S6c (Supporting Information), the bimerons in two sublattices both favor the anticlockwise chirality, namely, such weak inter‐sublattice interaction is sufficiently enough to unify the chirality of spin textures of the two sublattices. Therefore, the inter‐sublattice interactions are important for reversing the chirality of the bimeron in sublattices.

**Figure 4:**
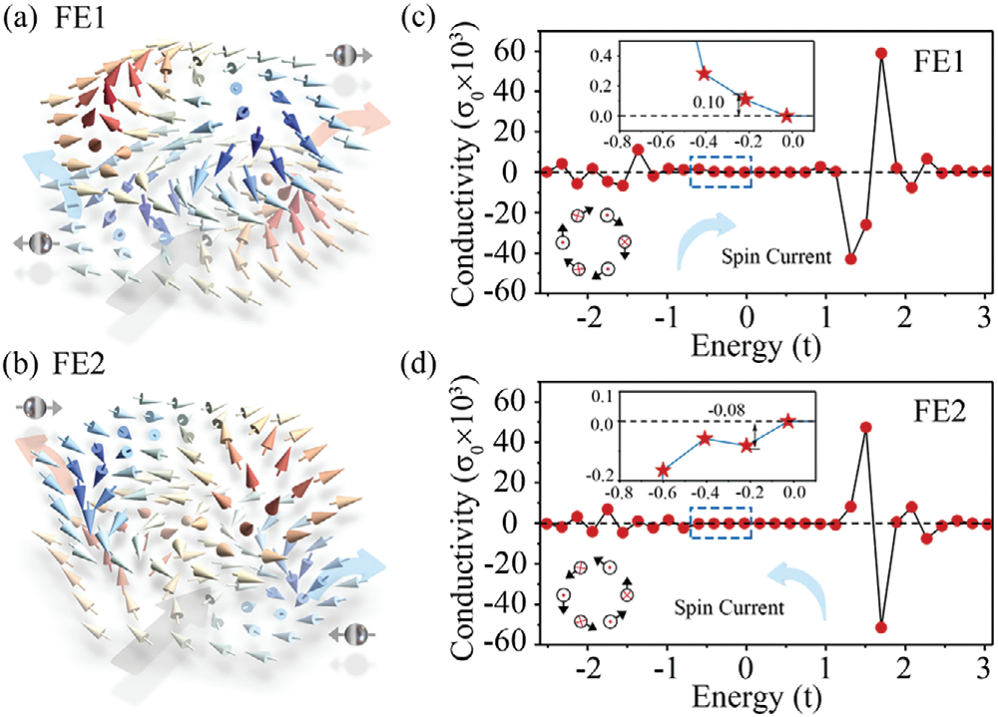
Schematic diagrams for the deflections of conduction electrons with σ_+_ and σ_−_ through AFM bimerons for a) FE1 and b) FE2. Red (blue) arrows indicate deflections of conduction electrons with σ_+_(σ_−_) in sublattice A(B). The gray arrow indicates the movement direction of AFM bimeron without the skyrmion Hall effect. Topological spin Hall conductivity for c) FE1 and d) FE2 of monolayer CuCr_2_Se_4_. Blue arrows indicate the deflection directions of spin currents for (c) FE1 and (d) FE2 around the Fermi level, respectively. In (c) and (d), σ_0_ =  e/4π and *t* = *t_2_* = 0.2 in Hamiltonian of the tight‐binding model.

Having established the strong coupling between DMI chirality, as well as AFM‐Q, and ferroelectricity in monolayer CuCr_2_Se_4_, we study the evolution of topological spin Hall conductivity under FE transition. We define the spins of conduction electrons along the +/− *x* axis as σ_+/−_ = +/− 1. According to our analysis above, for FE1 state with AFM‐Q = −1, the equivalent magnetic field of $B_{\sigma_{+}}^{A}$($B_{\sigma_{-}}^{B}$) exerted on conduction electrons along the +*z* (−*z*) axis direction. Therefore, the conduction electrons with σ_+_ experience a rightward transverse velocity when passing through the AFM bimeron in monolayer CuCr_2_Se_4_ and those with σ_−_ experience a leftward one, as shown in **Figure** **4**a. When transforming into FE2, the AFM‐Q is changed to +1 and the $B_{\sigma_{+}}^{A}$($B_{\sigma_{-}}^{B}$) is reversed to opposite direction. As a result, the conduction electrons with σ_+_ undergoes a leftward deflection and the conduction electrons with σ_−_ deflects in the opposite direction, as shown in Figure 4b. Then, by introducing the spin texture of monolayer CuCr_2_Se_4_ into the tight‐binding model, we calculate the topological spin Hall conductivity for FE1 and FE2. As shown in Figure 4c,d, the sign of spin Hall conductivity indeed is reversed under FE transition. Therefore, the ferroelectric control of TSHE is realized in monolayer CuCr_2_Se_4_.

At last, we wish to emphasize that the alter‐chirality of DMI for the multiferroic lattice is essential for realizing this mechanism. It enables the dominant DMI to be transferred to another sublattice under ferroelectric reversal, thereby realizing the reversal of the DMI chirality. The mechanism also requires the multiferroic lattice, the presence of AFM bimeron, and the relatively weak correlation between two sublattices. These ingredients limit the potential systems for the realization of TSHE to materials with a similar structure to CuCr_2_Se_4_, such as AgCr_2_X_4_, NaCr_2_X_4_ (X = S, Se), and so on.^[^ ^46^, ^47^ ^]^ Remarkably, these materials have been synthesized and extensively studied in recent years,^[^ ^41^, ^46^, ^47^, ^48^ ^]^ thereby rendering controlled TSHE promising in experimental research.

## 3 Conclusion

In conclusion, we propose a novel mechanism for realizing the effective control of TSHE in a controllable and reversible fashion. Our symmetry and model analysis shows that, for antiferromagnetic bimerons in the multiferroic lattice, the antiferromagnetic topological charge and Lorentz forces acted on conduction electrons can couple to DMI chirality, which enables the ferroelectric control of topological spin Hall conductivity and thus TSHE. Using first‐principles calculations and atomic spin model simulations, we further validate this mechanism in the experimentally feasible system of monolayer CuCr_2_Se_4_, which possesses alter‐chiral DMI. The alter‐chirality of DMI for the multiferroic lattice is revealed to be essential for realizing this mechanism.

## Conflict of Interest

The authors declare no conflict of interest.

## Supporting information



Supporting Information

## Data Availability

The data that support the findings of this study are available in the supplementary material of this article.
