# Supplementary material for: Engineering Topological Spin Hall Effect in 2D Multiferroic Material
Source: Adv Sci (Weinh). 2024 Oct 1;11(44):2407982. doi: 10.1002/advs.202407982 (PMC11600219; doi:10.1002/advs.202407982)
Supplement: Supplementary file 1 — Supporting Information [file ADVS-11-2407982-s001.docx]

Supporting Information

Engineering topological spin Hall effect in 2D multiferroic material

Kaiying Dou, Zhonglin He, Jiangyu Zhao, Wenhui Du, Ying Dai^*^, Baibiao Huang, Yandong Ma^*^

**Note S1: Methods**

Our first-principles calculations are performed based on density functional theory (DFT) as implemented in the Vienna ab initio Simulation Package (VASP).^[1,2]^ The generalized gradient approximation (GGA) in the form of Perdew-Burke-Ernzerhof (PBE) is used to treat the exchange-correlation interaction.^[3]^ The cutoff energy is set to 520 eV. The convergence criteria for the energy and residual force are set to 10^-6^ eV and 0.01 eV/Å, respectively. The vacuum space along the z direction is set to 30 Å to avoid adjacent interactions. Atom coordinates are fully relaxed with the Γ-centered Monkhorst-Pack scheme of $7\times7\times1$ k-point mesh.^[4]^ Configuration- and chirality-dependent total energy difference methods are used for obtaining exchange coupling strength and DMI strength, which are successfully employed for magnetic parameter calculations.^[5,6]^ For exchange interaction parameter calculations, a $1\times2\sqrt{3}$ supercell with a k-point mesh of $20\times5\times1$ is adopted, and a $5\times20\times1$ k-point mesh is employed for $4\times1$ supercell to calculate DMI parameters. To describe the strong correlations of Cr-3*d* electrons, GGA+U method with effective Hubbard U＝5 eV is adopted , which is consistent with the previous work.^[7]^

The atomic spin model simulations are performed using the VAMPIRE package based on the atomic spin Hamiltonian and Landau-Lifshitz-Gilbert (LLG) equation.^[8-10]^ All simulations are gradually cooled down from initial disordered state at high temperature ($T \gg T_{c}$) to 0 K. The stable spin textures are obtained using a $240\times240\times1$ supercell with periodic conditions.

**Note S2: Motion of current driven topological spin textures**

The motion of current driven topological spin texture is characterized by Thiele’s equation ^[11,12]^

$$\boldsymbol{G}\times\boldsymbol{v}-\alpha\mathcal{D}\cdot\boldsymbol{v}+4\pi\mathcal{\mathcal{B}\cdot}\boldsymbol{j}=0$$

Here, $\boldsymbol{G}=\left( 0, 0, -4\pi Q \right)$ is the gyromagnetic coupling vector, $\alpha$ is the magnetic damping parameter and $\mathcal{D}=4\pi\left( \begin{matrix} D_{ii} & D_{ij} \\ D_{ji} & D_{jj} \end{matrix} \right)$ is the dissipative force tensor with $D_{ij}=\frac{1}{4\pi}\int\partial_{\boldsymbol{i}}\boldsymbol{m}\cdot\partial_{\boldsymbol{j}}\boldsymbol{m}dxdy$. The spin Hall tensor $\mathcal{\mathcal{B}}=B_{0}\left( \begin{matrix} H_{ii} & H_{ij} \\ H_{ji} & H_{jj} \end{matrix} \right)$ describes the efficiency of spin-orbit torque over topological spin texture. $\boldsymbol{v}=(v_{x},v_{y})$ is the propagation velocity of topological magnetism. The velocity components can be obtained by Thiele’s equation as $v_{x}=[-\alpha\mathcal{D}/{(Q}^{2}+{\alpha^{2}\mathcal{D}}^{2})]\mathcal{\mathcal{B}}j$ and $v_{y}=[Q/{(Q}^{2}+{\alpha^{2}\mathcal{D}}^{2})]\mathcal{\mathcal{B}}j$. Among them, the opposite Q result in the opposite transverse motion $v_{y}$ of topological spin texture.

**Note S3：The model of interaction between Lorentz force and conduction electrons in AFM bimeron**


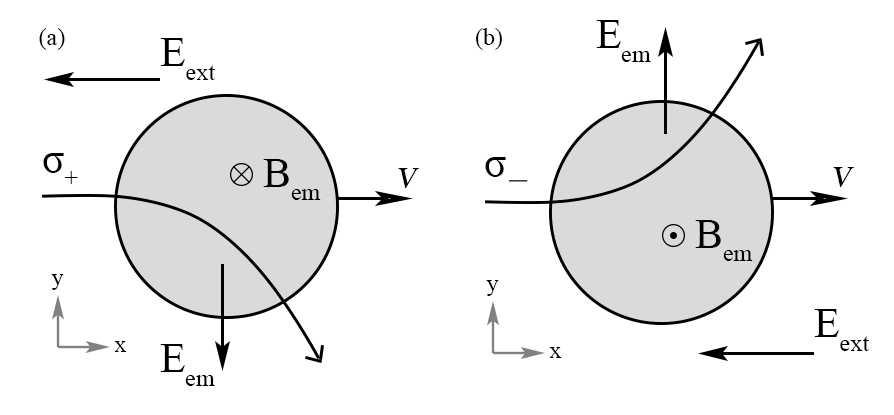


**Fig. S1** Diagrams of the emergent electromagnetic field on a AFM bimeron when a current passes through it.

To get more insight into this mechanism, we employ a model for describing interaction between Lorentz force and conduction electrons in AFM bimeron. When applying an external electric field along -x direction, the conduction electrons in AFM bimeron would move along x direction. During the process, spin-up electrons $\sigma_{+}$ (spin-down electrons $\sigma_{-}$) in sublattice A (B) are deflected by the force of the emergent magnetic field $\boldsymbol{B}_{\sigma_{+}}^{A}= -\frac{\hbar}{2e}N_{x,y}(\boldsymbol{r})\boldsymbol{z}$ [$\boldsymbol{B}_{\sigma_{-}}^{B}= \frac{\hbar}{2e}N_{x,y}(\boldsymbol{r})\boldsymbol{z}$], as shown in **Fig. S1**. In this regard, the Lorentz forces of $\boldsymbol{F} = e\left( \dot{\boldsymbol{r}}\times\boldsymbol{B}_{em} \right)$ acted on conduction electrons in such AFM bimeron are determined by $N_{x,y}(\boldsymbol{r})$.

The motion of the AFM bimeron causes an emergent electric field in the direction perpendicular to the motion of bimeron as shown in **Fig. S1**. Given that electrons are in motion at a considerably faster pace than AFM bimeron, the latter is effectively stationary with regard to the electrons.^[13]^ Consequently, the impact of the emergent electric field on the electron deflection is nearly negligible and the Lorentz force $\boldsymbol{F} = e\left( \boldsymbol{E}_{\mathrm{em}}+\dot{\boldsymbol{r}}\times\boldsymbol{B}_{\mathrm{em}} \right)$ can be written as $\boldsymbol{F} = e\left( \dot{\boldsymbol{r}}\times\boldsymbol{B}_{\mathrm{em}} \right)$.

**Note S4：Two strategies of reversing AFM-Q for AFM bimeron**

For convenience of discussion, we employ a concise representation that solely indicates the variations in *x* and *y* directions. This approach effectively captures the unique characteristics of a topological magnetic texture. The antiferromagnetic (AFM) bimeron consists of two ferromagnetic bimerons with opposite magnetization. Therefore, we take sublattice A as an example and sublattice B can be derived from $\boldsymbol{M}_{A}\boldsymbol{(r)= -}\boldsymbol{M}_{B}\boldsymbol{(r)}$.


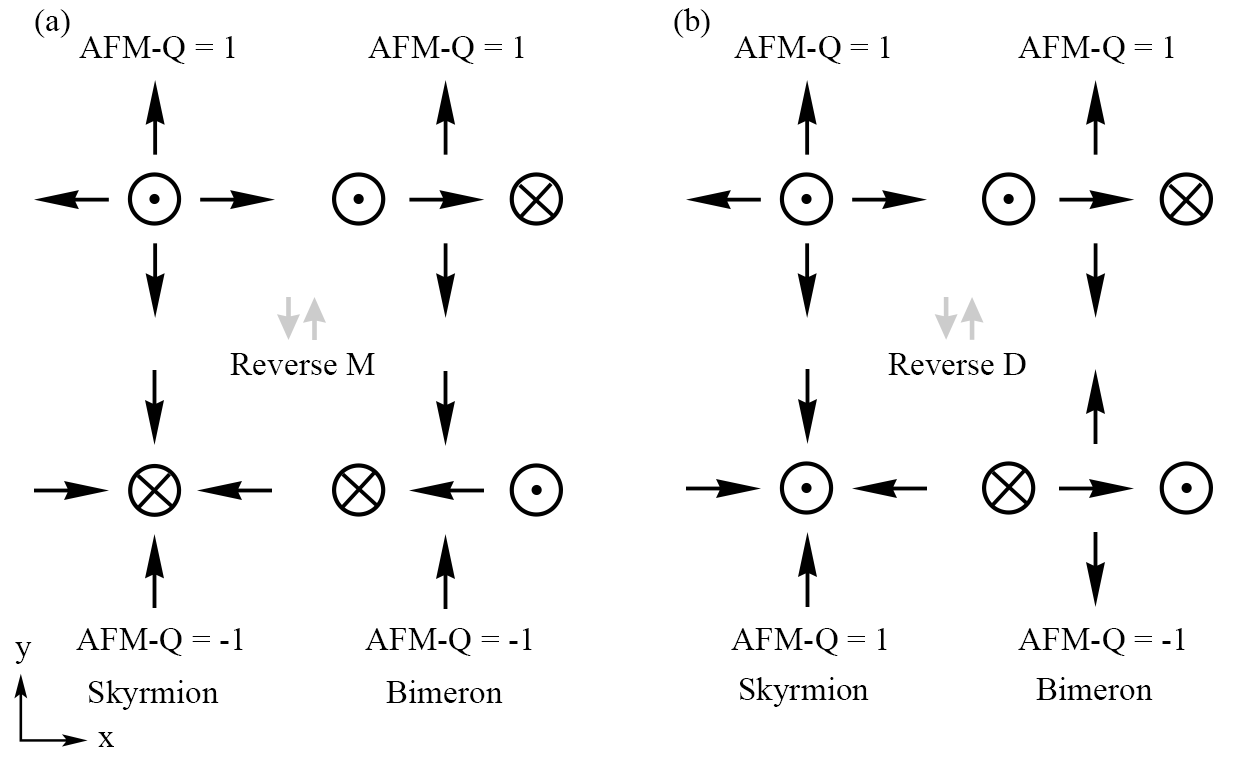


**Fig. S2** (a) Schematic diagrams of spin orientation reversal for AFM skyrmion and bimeron, as well as their AFM-Q. (a) Schematic diagrams of DMI reversal for AFM skyrmion and bimeron, as well as their AFM-Q.

**Note S5：Expressions of AFM bimeron and analysis of AFM-Q.**

For convenience of discussion, we employ a concise representation that solely indicates the variations in *x* and *y* directions. This approach effectively captures the unique characteristics of a topological magnetic texture. The AFM bimeron consists of two ferromagnetic bimerons with opposite magnetization. Therefore, we take sublattice A as an example and sublattice B can be derived from $\boldsymbol{M}_{A}\boldsymbol{(r)= -}\boldsymbol{M}_{B}\boldsymbol{(r)}$. A bimeron can be conceptualized as arising from a π/2 rotation of each spin in a skyrmion around a certain axis. Considering a skyrmion $\boldsymbol{m}(\boldsymbol{r}) = (sin r\cdot cos \theta, sin r\cdot sin \theta, cos r)$ shown in the left panel of **Fig. S3a**, the bimeron $\boldsymbol{m}(\boldsymbol{r}) =( cos r, sin r\cdot sin \theta, -sin r\cdot cos \theta)$ can be obtained by a clockwise rotation around *y*-axis by π/2, as shown in the right panel of **Fig. S3a**.


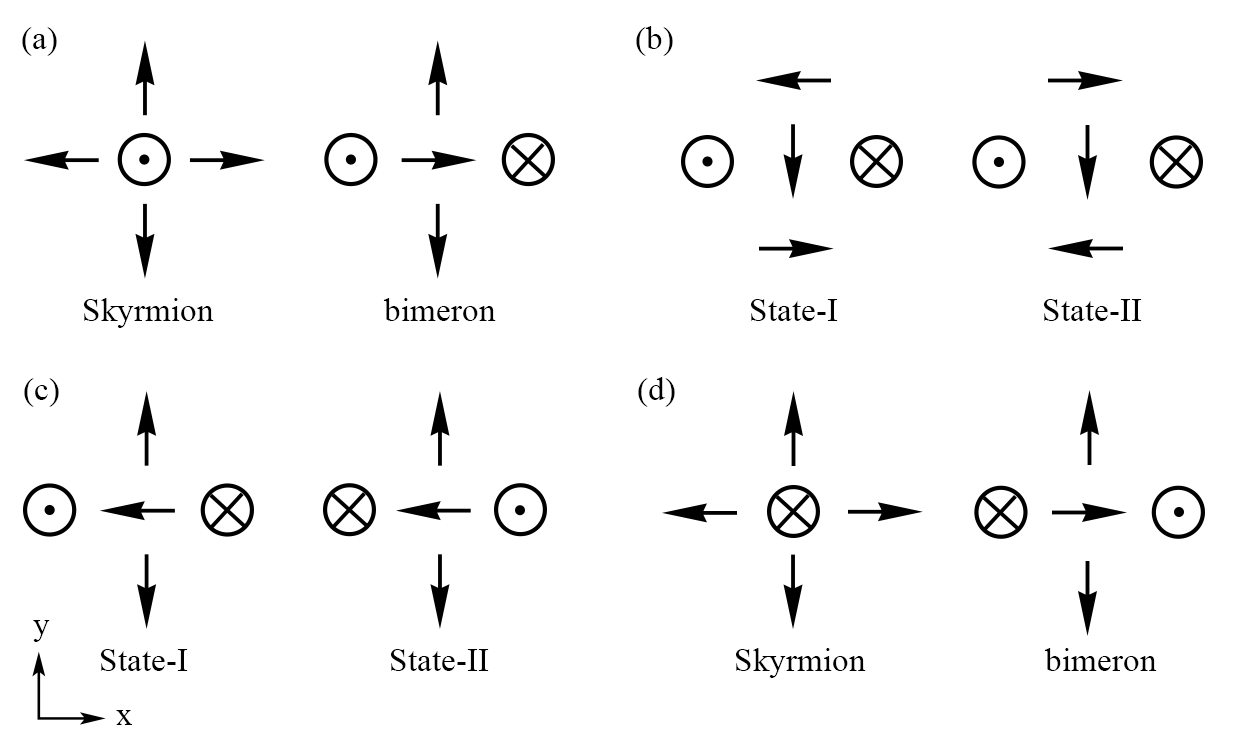


**Fig. S3** Schematic diagrams of (a) topologically equivalent skyrmion and bimeron, (b) two states of bimeron controlled through reversing DMI chirality in monolayer VOI_2_,^[14]^ (c) two states of bimeron for monolayer CuCr_2_Se_4_. (d) Topologically equivalent skyrmion and bimeron, with opposite polarity and DMI chirality compared to (a), respectively.

To investigate the expressions of reversing DMI chirality, we need to discuss the specific textures of bimeron with opposite chirality of DMI. Xu *et. al.* reported the bimeron controlled through the mediation of DMI chirality in monolayer VOI_2_,^[14]^ as shown in **Fig. S3b**. The direction of DMI is out-of-plane and thus reversing the DMI only alters the component of each spin along *x* axis. In our work, the sublattice A for FE1 and FE2 states are shown in **Fig. S3c**. When reversing the DMI chirality, in-plane DMI results in the reversal of spin component along the *z* axis. Therefore, the variation of bimeron under DMI reversal relied on the DMI direction of a given system. The expression used in the main text corresponds to the case of in-plane DMI.

We further discuss the effect of reversing DMI chirality on the topological charge in bimeron. As the topological charge represents the character of topology in bimeron, it cannot be changed by a continuous transformation of magnetization.^[15,16]^ Based on it, the bimeron derived from rotating spins of skyrmion is topological equivalent to its skyrmion counterpart. When reversing the DMI chirality of bimeron shown in the right panel of **Fig. S3a**, we obtain the bimeron shown in the right panel of **Fig. S3d**. They originate from the spin rotations of two skyrmions with opposite polarities shown in the left panels of **Fig. S3a** and **d**. Therefore, reversing DMI chirality in bimeron can be equivalently considered as reversing the polarity in skyrmion, leading to a change of topological charge. However, the DMI chirality of skyrmions is not related to the sign of topological charge and thus cannot be regulated by ferroelectricity.^[14]^ Therefore, the bimerons are chosen in our work instead of skyrmions.

**Note S6: Heisenberg exchange interactions**

For determining magnetic interactions shown in **Fig. S4**, we consider eight magnetic structures, i.e., *FM* ($++++++++$), *AFM1* ($+-+-+-+-$), *AFM2* ($+--++--+$), *AFM3* ($++--++--$), *AFM4* ($+-+-++--$), *AFM5* ($++-+++--$), *AFM6* ($+-+++-++$), *AFM7* ($+-++-+--$).^[17]^


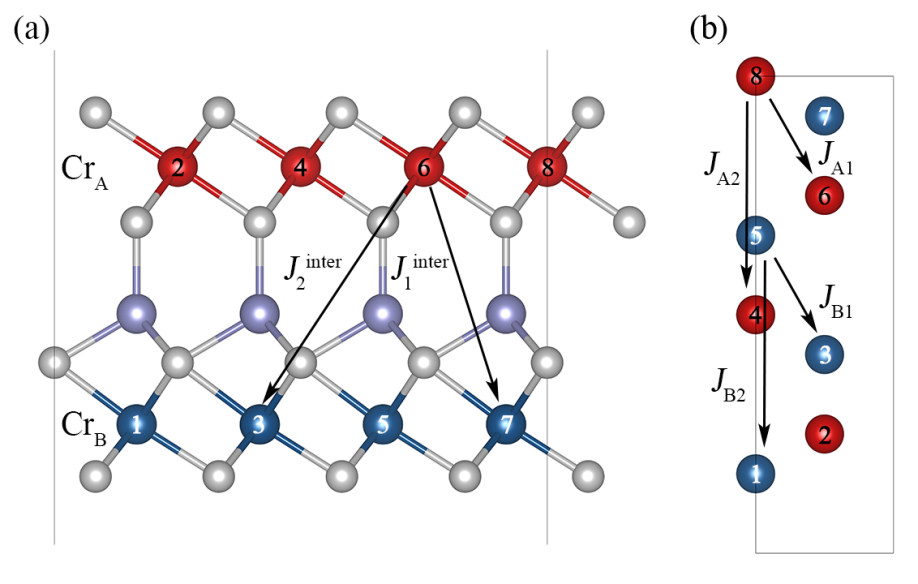


**Fig. S4** Schematic diagrams for different Heisenberg exchange interactions in 2D atomic spin Hamiltonian model. (a) The side view and (b) the top view of $1\times2\surd3$ supercell of the monolayer CuCr_2_Se_4_ are illustrated.

**Note S7: Spin textures for FE1 and FE2 states of monolayer CuCr_2_Se_4_**

**
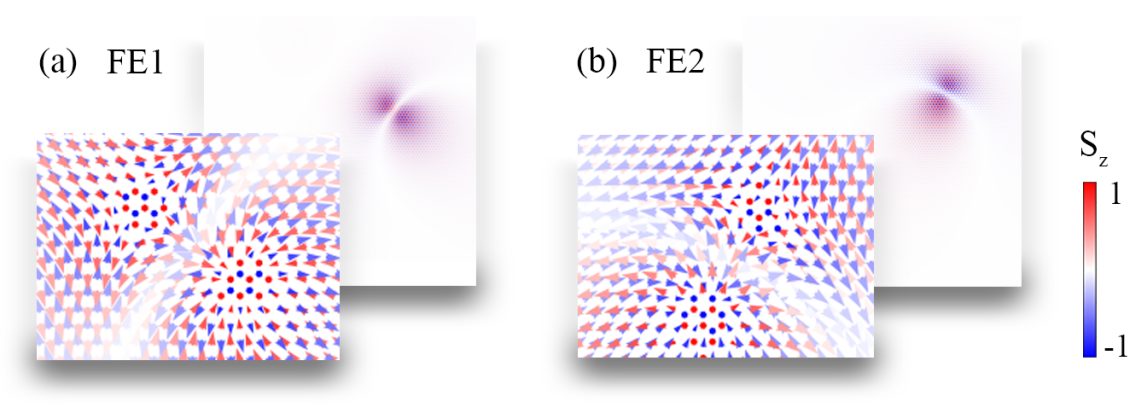
**

**Fig. S5** Specific spin patterns for (a) FE1 and (b) FE2 states of monolayer CuCr_2_Se_4_. Color map specifies the out-of-plane spin component and arrows indicate the local in-plane spin component.

**Note S8: Inter-sublattice coupling**

**
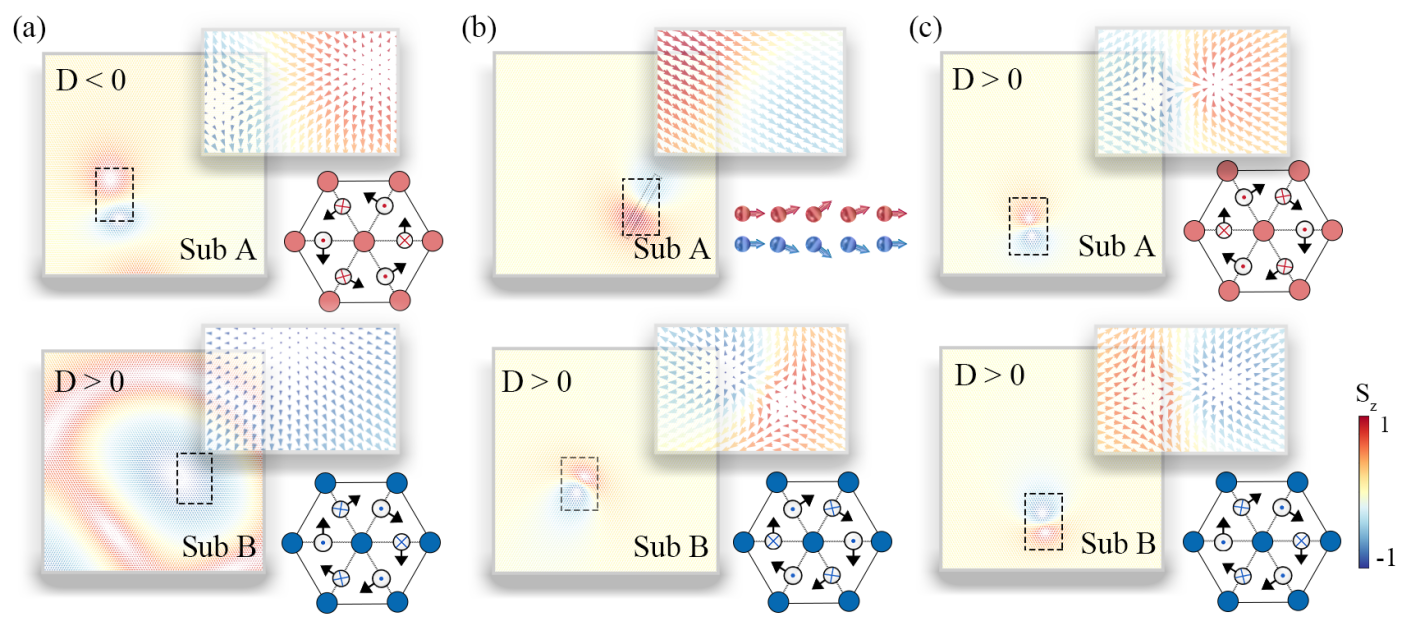
**

**Fig. S6** Specific spin patterns for the monolayer CuCr_2_Se_4_ (a) without inter-sublattice interactions, and with the inter-sublattice interaction reduced to (b) 1/10 and (c) half of original strength. Color map specifies the out-of-plane spin component and arrows indicate the local in-plane spin component.

**Note S9: Topological charge Q.**

The topological charge in ferromagnetic system can be described as ^[18]^

$$Q=\frac{1}{4\pi}\int\boldsymbol{m}\cdot\left( \frac{\partial\boldsymbol{m}}{\partial x}\times\frac{\partial\boldsymbol{m}}{\partial y} \right)dxdy,$$

where ***m*** is the normalized local magnetic moments of discussed spin textures. For meron and antimeron, Q = $\pm$1/2. Bimeron consists of a meron and an antimeron with same topological charge.^[13]^ Therefore, Q of bimeron is $\pm$1. In AFM system, the AFM-Q is defined as a similar expression except for the meaning of ***m***. The AFM spin texture ***m*** can be described as the normalized staggered order vector of $\boldsymbol{m(r)}=[\boldsymbol{M}_{A}\boldsymbol{(r)}-\boldsymbol{M}_{B}\boldsymbol{(r)}]/2$, where $\boldsymbol{M}_{A}\boldsymbol{(r)}$ and $\boldsymbol{M}_{B}\boldsymbol{(r)}$ are the normalized local magnetic moments of sublattice A and B, respectively.

**References**

1. S. Grimme, J. Antony, S. Ehrlich, K. Krieg, A consistent and accurate ab initio parametrization of density functional dispersion correction (DFT-D) for the 94 elements H-Pu, J. Chem. Phys. 132, 154104 (2010).
2. G. Kresse, J. Furthmuller, Efficient iterative schemes for ab initio total-energy calculations using a plane-wave basis set, Phys. Rev. B 54, 11169-11186 (1996).
3. J. Perdew, K. Burke; M. Ernzerhof, Generalized gradient approximation made simple, Phys. Rev. Lett. 77, 3865-3868 (1996).
4. H. Monkhorst, J. Pack, Special points for Brillouin-zone integrations, Phys. Rev. B 13, 5188 (1976).
5. H. Yang, A. Thiaville, S. Rohart, A. Fert, M. Chshiev, Anatomy of Dzyaloshinskii- Moriya interaction at Co/Pt interfaces, Phys. Rev. Lett. 115, 267210 (2015).
6. M. Soumyanarayanan, A. Gonzalez Oyarce, A. Tan, M. Im, A. Petrovic, P. Ho, K. Khoo, M. Tran, C. Gan, F. Ernult, C. Panagopoulos, Tunable room-temperature magnetic skyrmions in Ir/Fe/Co/Pt multilayers, Nat. Mater. 16, 898-904 (2017).
7. T. Zhong, X. Li, M. Wu, J. Liu, Room-temperature multiferroicity and diversified magnetoelectric couplings in 2D materials, Nat. Sci. Rev. 7, 373-380 (2020).
8. VAMPIRE software package version 5.0 available from https://vampire.york.ac.uk.
9. R. Evans, W. Fan, P. Chureemart, T. Ostler, M. Ellis, R. Chantrell, Atomistic spin model simulations of magnetic nanomaterials, J. Phys.: Condens. Matter 26, 103202 (2014).
10. P. Asselin, R. Evans, J. Barker, R. Chantrell, R. Yanes, O. Chubykalo-Fesenko, D. Hinzke, U. Nowak, Constrained Monte Carlo method and calculation of the temperature dependence of magnetic anisotropy, Phys. Rev. B 82, 054415 (2010).
11. W. Jiang, X. Zhang, G. Yu, W. Zhang, X. Wang, M. Jungfleisch, J. Pearson, X. Cheng, O. Heinonen, K. Wang, Y. Zhou, A. Hoffmann, S. Velthuis, Direct observation of the skyrmion Hall effect. Nat. Phys. 13, 162–169 (2017).
12. Thiele, A. Steady-state motion of magnetic domains. Phys. Rev. Lett. 30, 230 (1973).
13. T. Lancaster, Skyrmions in magnetic materials, Contemp. Phys. 60, 246-261 (2019).
14. C. Xu, P. Chen, H. Tan, Y. Yang, H. Xiang, L. Bellaiche, Electric-field switching of magnetic topological charge in type-I multiferroics, Phys. Rev. Lett. 125, 037203 (2020).
15. W. Du, K. Dou, Z. He, Y. Dai, B. Huang, Y. Ma, Spontaneous magnetic skyrmions in single-layer CrInX_3_ (X = Te, Se), Nano Lett. 22, 3440-3446 (2022).
16. Z. He, K. Dou, W. Du, Y. Dai, B. Huang, Y. Ma, Mixed Bloch-Néel type skyrmions in a two-dimensional lattice, Phys. Rev. B 109, 024420 (2024).
17. Q. Cui, Y. Zhu, J. Liang, P. Cui, H. Yang, Antiferromagnetic topological magnetism in synthetic van der Waals antiferromagnets, Phys. Rev. B 107, 064422 (2023).
18. B. Berg, M. Luscher, Definition and statistical distributions of a topological number in the lattice O(3) σ-model, Nuclear Phys. B 190, 412-424 (1981).
